# Optimization of terahertz broadband polarization insensitive metamaterial absorber using bisecting rectangles technique based on quadratic surrogates

**DOI:** 10.1038/s41598-025-32419-7

**Published:** 2026-01-22

**Authors:** Ahmed S. Etman, Mohamed Farhat O. Hameed, S. S. A. Obayya, Ahmed E. Hammad

**Affiliations:** 1 Department of Engineering Mathematics and Physics, Faculty of Engineering, Cairo University, Giza, 12613 Egypt; 2 Nanoelectronics Integrated Systems Center, Nile University, Giza, 12588 Egypt; 3 Centre for Photonics and Smart Materials, Zewail City of Science, Technology and Innovation, October Gardens, 6th of October City, Giza 12578 Egypt; 4 Centre of Nanotechnology, Zewail City of Science, Technology and Innovation, October Gardens, 6th of October City, Giza 12578 Egypt; 5 Nanotechnology and Nanoelectronics Engineering Program, Zewail City of Science, Technology and Innovation, October Gardens, 6th of October City, Giza 12578 Egypt; 6 Mathematics and Engineering Physics Department, Faculty of Engineering, University of Mansoura, Mansoura, 35516 Egypt

**Keywords:** Engineering, Materials science, Optics and photonics, Physics

## Abstract

In this paper, an optimization algorithm based on bisecting hyperrectangles and constructing quadratic models is exploited to attain the optimal design of a broadband metamaterial absorber (MMA) with small thickness and high absorption in the terahertz (THz) frequency range. The full wave vectorial finite element method is employed along with the introduced algorithm to simulate and optimize the investigated design. The presented absorber comprises of a vanadium dioxide (VO_2_) metallic layer placed on a lossy polyimide dielectric substrate. Further, the reported design exhibits a broad bandwidth of 2.9 THz where the absorptivity exceeds 90% through a frequency range between 2.43 and 5.33 THz. In addition, the proposed design could obtain polarization insensitive absorption spectrum for the transverse electric (TE) as well as transverse magnetic (TM) polarizations. Furthermore, the absorption spectrum of the suggested absorber could realize high absorptivity (greater than 80%) for the TE and TM polarizations when the wave incident angle is varied from normal incidence to 50°. Therefore, the reported optimization algorithm is highly recommended for THZ broadband MMAs based applications.

## Introduction

Metamaterials have aroused great research interest in the few past years. Such periodic structures possess many distinctive and exotic electromagnetic characteristics that cannot be found in natural materials, such as negative refractive indices^1^, invisibility cloaking^2^, superlunary^3^‎‎, asymmetric transmission^4^, and imaging^5^. All of these advantageous and extraordinary features make the metamaterials become great candidates in a wide range of applications such as modulators^6^, cross polarization conversion^7^, sensors^8^, communication^9^, switches^10^, filters^11^, power detectors^12^, solar energy harvesting^13^, and EM wave absorption^14^.

Recently, terahertz (THz) technology within the frequency band between 0.1 THz and 10 THz has earned a great concern due to its applications in different fields^15,16^. Further, the progress in most of these applications is highly dependent on terahertz absorbers. However, the effective absorption in the THz band using the naturally existing materials is a great challenge. Therefore, the attributes of metamaterials have been explored for the precise design of THz metamaterial absorbers (MMAs)^17,18^. The performance of such absorbers has surpassed the conventional electromagnetic absorbers^19,20^ due to their outstanding traits such as small thickness, flexible and easily fabricable design with low fabrication cost, high absorption efficiency, and a tunable absorption spectrum. The first perfect metamaterial narrowband absorber was experimentally suggested by Landy et al.^21^ in 2008. Subsequently, several studies on MMAs^17,18,22,23^ were performed to achieve perfect absorption spectrum with high absorptivity, polarization independence and robust performance for oblique incident waves. However, the resonance nature of most of the previously introduced absorbers results in limited bandwidth designs that cannot be used in broadband applications. Therefore, various approaches are investigated to enhance the bandwidth of the proposed absorber. In this context, several resonators have been combined with distinct geometries over a single unit cell^24^ to achieve broad band absorption. Additionally, resonators based on multiple layers have been used separated by dielectric spacers^25^. Nevertheless, such techniques lead to large unit cell size and complex fabrication process. Thus, developing a simple and easily fabricable broadband MMA is a problem with a great challenge.

Several THz MMAs have been reported in the literature^26–34^ to achieve broadband absorption. In^26^, a broadband MMA with a tunable absorption was investigated. The presented design achieved higher absorption than 90% in the frequency band from 3.287 THz to 5.247 THz. Also, two tunable broadband absorbers with graphene resonators were demonstrated in^27^. The first MMA exhibited high absorptance through a frequency range from 2.06 to 4.24 THz. The absorptance of such design still exceeded 72% and 90% for TE and TM polarizations, respectively for different values of incident angles (less than 50°). Moreover, the second THz MMA in^27^ was obtained by adding a polyimide layer above the resonator of the previous absorber structure. This improved the bandwidth of the suggested design to be 3.14 THz instead of 2.18 THz. Further, an asymmetric square ring structure was proposed in^28^ which achieved high absorptivity greater than 90% between 0.98 and 1.63 THz with a total bandwidth of 0.65 THz. The investigated design can still maintain the absorptivity more than 80% over a wide angular range up to 50°. In addition, Wu et al.^29^ suggested a bi-channel switchable broadband MMA based on a VO_2_ patch. Such a design has six layers and could achieve two broadband absorption windows with a high absorption performance exceeding 90%. The first design exhibits a bandwidth covering the frequency band between 1.31 and 3.18 THz when the VO_2_ is in dielectric state. However, the absorption window moves to the frequency band from 4.45 to 8.4 THz when VO_2_ is in the metallic state. Besides, a broadband THz MMA with a disk-shaped graphene structure was proposed in^30^. The introduced design could provide an absorption exceeding 90% over a frequency bandwidth of 2.173 THz regardless the polarization of the incident wave with a good tolerance to the wave incident angle (up to 30°). Sabaruddin et al. ^31^ have also studied a wide band MMA using gold and graphene patterned metasurfaces with a good absorption spectrum exceeding 80% between the frequencies 0.95 and 1.95 THz and a maximum absorption value of 98.9%. Furthermore, a polarization insensitive THz MMA consists of four layers with square shapes is suggested in^32^ with high absorptivity through the frequency range from 1.45 to 4.35 THz. The absorption of this absorber was obtained over a wide angular range (up to 40° for TE polarization and up to 45° for TM polarization). Also, Niu et al.^33^ have investigated a six layers MMA based on a VO_2_ resonator with high absorptivity exceeding 90% over the frequency range between 3.33 and 5.62 THz. The reported design can keep the absorptivity higher than 80% for the two polarized incident waves when changing the wave incident angle up to 60°. Recently, a five layers structure was investigated using graphene and VO_2_ layers by Wang et al.^34^. Such absorber could realize a broadband bandwidth of 2.24 THz.

It should be noticed that the optimal values of the geometrical design parameters of most of the previously introduced THz MMAs are obtained through performing a parametric sweep investigation where the impact of varying only single design parameter on the absorption spectrum is separately studied while the other geometrical parameters are kept at constant values. This approach narrows the design space and yields only locally optimal geometric parameters. In order to obtain the global optimum an optimization algorithm is highly needed that explores the entire design space.

Various metaheuristic optimization algorithms have been previously used in the design and the optimization of THz MMAs in the literature^35,36^. However, these optimization algorithms often converge to solutions that are only locally superior to nearby candidates, rather than to the global optimum^37,38^. In addition, the optimal design found depends on the chosen initial values of the design geometrical parameters^37^. In this work, a global optimization technique that bisects the space into hyperrectangles^39^ is combined with a quadratic surrogate model^40^ to design a broadband THz MMA with high absorption response over a wide range of THz frequencies. The fast convergence rate of the introduced optimization algorithm to the global optimal geometric parameters is guaranteed^39^. Further, the algorithm does not need to evaluate or approximate the optimized function derivatives. In addition, a quadratic model^40^ is exploited and optimized instead of the high-fidelity optimized function to reduce the time wasted in calculating the optimized function with high accuracy. The introduced optimization technique is combined with the frequency domain solver in the CST software package^41^ to find the global optimal values of the design parameters of the novel proposed broadband absorber. The suggested design could achieve high absorption response exceeding 90% over a broad frequency range between 2.43 and 5.33 THz. Also, the absorption performance for TE and TM polarizations coincide which results in a polarization insensitive design. Moreover, the presented structure could maintain the absorptivity higher than 80% for various angular range (up to 50°). Furthermore, a comparative study between the investigated absorber and some previous THz MMAs^26–34^ is also reported to illustrate the power and strength of the introduced optimization algorithm in achieving a flexible simple design, polarization insensitive broadband THz MMA that can be fabricated with a compact size and a small thickness. As a result, the proposed MMA is a potential candidate in several THz applications.

### Bisecting rectangles optimization technique

A global optimization technique that bisects the design space to smaller hyperrectangles (BIRECT)^39^ is introduced. In this algorithm, a normalization in each direction is initially performed to describe the design space as a unit hypercube. Subsequently, smaller hyperrectangles are obtained through a partitioning strategy for the design space and the optimized function is evaluated at the diagonal points of these hyperrectangles. Finally, the global optimal point is obtained by choosing the minimum optimized function value. Further, the presented technique builds quadratic surrogates^40^ to replace the intensive CPU to save the optimization process time.

The studied optimization problem takes the form

1 $$\:{}_{\boldsymbol{x}\:\in\:\boldsymbol{F}\:}{}^{min\:}m\left(\boldsymbol{x}\right)$$

where$\:\:\:\boldsymbol{F}=\left\{\boldsymbol{x}\in\:\:{\mathbb{R}}^{\boldsymbol{n}}|\:{l}_{i}\le\:\:{x}_{i\:}\le\:\:{u}_{i\:},\:\:i=\mathrm{1,2},\dots\:..n\:\:\right\}\:$is the design space, $\:n$ is its dimension and $\:m\left(\boldsymbol{x}\right)$is the objective function. In the proposed technique, a unit hypercube $\:\boldsymbol{H}$ will be used to represent the design space $\:\boldsymbol{F}\:$which leads to the following optimization problem

2 $$\:{}_{\boldsymbol{x}\:\in\:\boldsymbol{H}}{}^{min\:}\:m\left(\boldsymbol{x}\right)$$

where $\:\:\boldsymbol{H}=\left\{\boldsymbol{x}\in\:\:{\mathbb{R}}^{\boldsymbol{n}}|0\le\:\:{x}_{i\:}\le\:1\:,\:\:i=\mathrm{1,2},\dots\:..n\:\:\right\}$. Initially, the optimized function $\:m\left(\boldsymbol{x}\right)$ is calculated at two points on the main diagonal of $\:\boldsymbol{H}$, namely $\:{p}_{1}=$ (1/3,1/3,…,1/3) and $\:{p}_{2}=$ (2/3,2/3,…,2/3). Consequently, the initial hypercube is divided into two hyperrectangles along the dimension whose index is the smallest one. Further, the optimization technique chooses some of the available hyperrectangles to be candidate for further divisions according to the following conditions^39^

3 $$\:min\:\left\{m\left({p}_{1}^{k}\right),\:m\left({p}_{2}^{k}\right)\right\}-\gamma\:{\:\sigma\:}_{k}\le\:min\:\left\{m\left({p}_{1}^{j}\right),\:m\left({p}_{2}^{j}\right)\right\}-\gamma\:{\:\sigma\:}_{j\:},\:\forall\:j\in\:J,$$

4 $$\:min\:\{m\left({p}_{1}^{k}\right),\:m\left({p}_{2}^{k}\right)\}-\gamma\:{\:\sigma\:}_{k}\le\:{m}_{\mathrm{min}}-\delta\:\left|{m}_{\mathrm{min}}\right|$$

where $\:\gamma\:$ is some positive scalar and $\:{p}_{1}^{j}$, $\:{p}_{2}^{j}$ are the points that divide the main diagonal of the $\:{j}^{th}$ hyperrectangle into three equal segments, $\:j\in\:J$ (the group of the all constructed hyperrectangle up to the current iteration), $\:{\sigma\:}_{j}$ refers to volume of the $\:{j}^{th}$ hyperrectangle, $\:{m}_{\mathrm{m}\mathrm{i}\mathrm{n}}$ is the smallest achieved objective function till the current state. Further, $\:\delta\:\:$is a scalar with a positive value which is used to prevent the further partitioning of the hyperrectangles that have small volume and optimal values of objective function. Therefore, the proposed algorithm does not waste most of the optimization time in the local search.

**Fig. 1:**
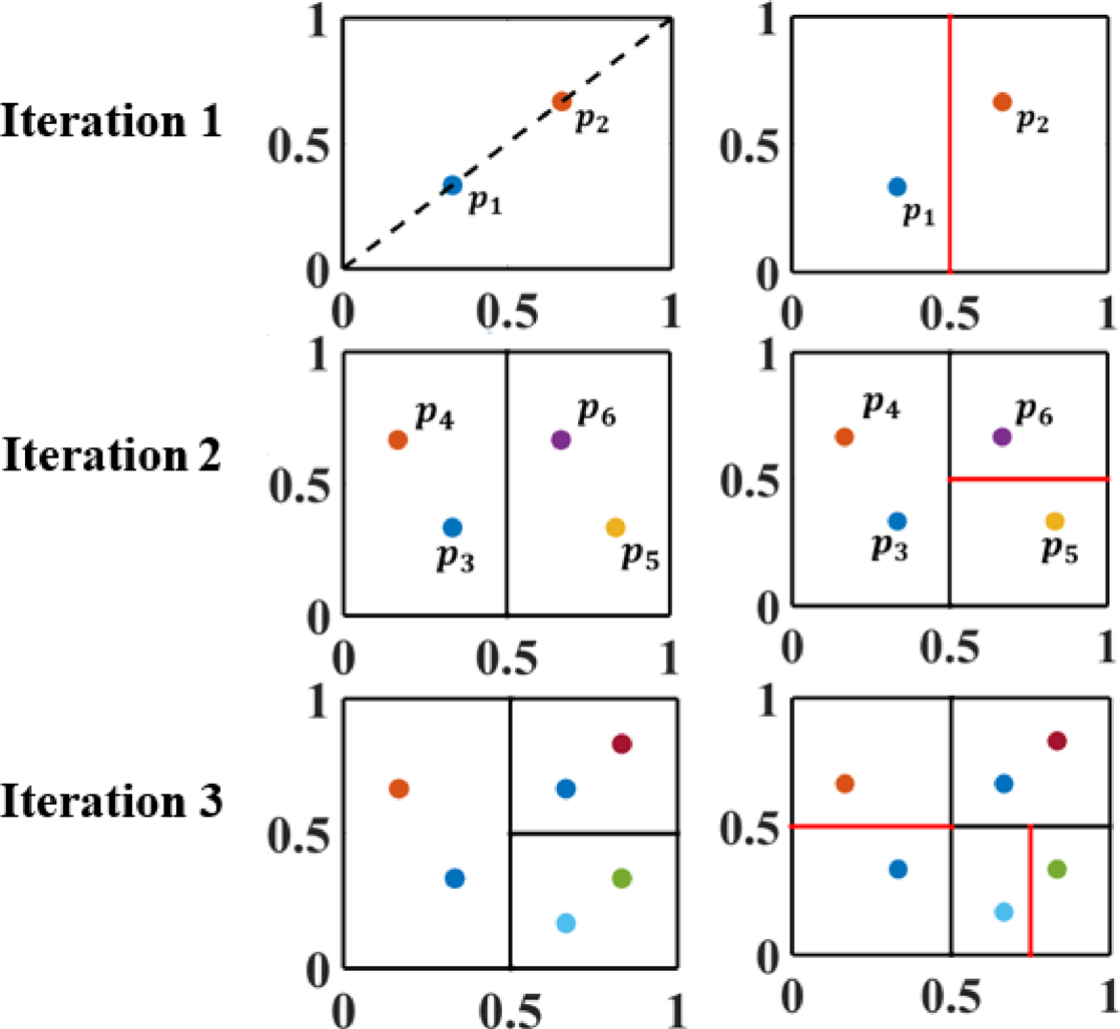
Demonstration of the BIRECT algorithm across three iterations for a two-dimensional example.

To demonstrate the procedure of the proposed algorithm, a 2D example will be presented. The design space is first normalized to a unit square, and the optimized function is computed at the two points $\:{p}_{1}=\left(\frac{1}{3},\frac{1}{3}\right)$ and at $\:{p}_{2}=\left(\frac{2}{3},\frac{2}{3}\right)$. Then, the square is bisected into two smaller rectangles along the x-direction (dimension with lowest index) as the square has two sides with the same length as shown in Fig. 1.

**Fig. 2:**
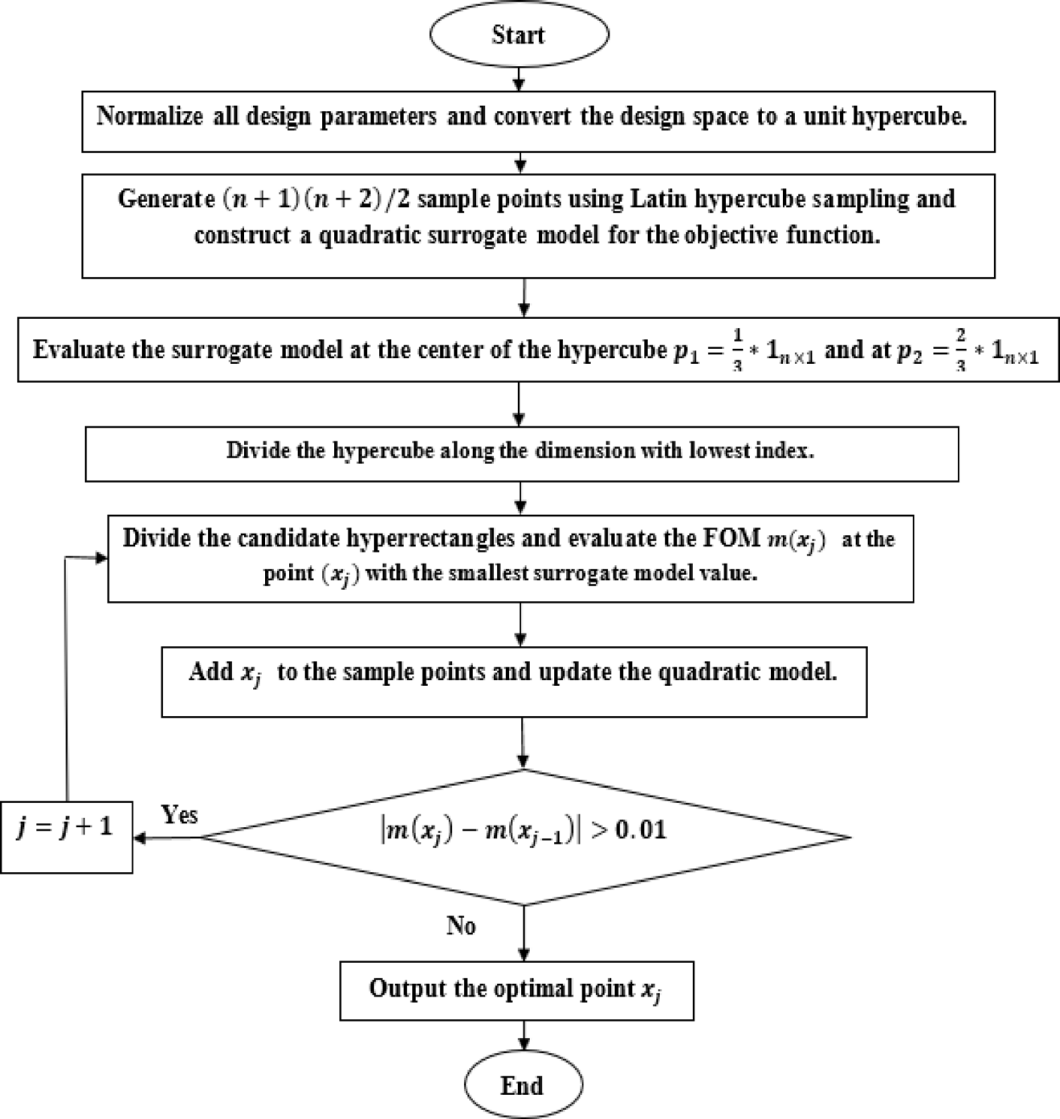
A flowchart of the presented optimization algorithm.

Subsequently, two points will be generated for each rectangle. For the left rectangle, $\:{p}_{3}$ will be kept the same as $\:{p}_{1}$ in the first iteration. However, the x-coordinate of the point $\:{p}_{4}$ is calculated by subtracting the length of left rectangle along the x-direction (direction of division in the first iteration) from the x-coordinate of the point $\:{p}_{2}$ while the y-coordinate of the point $\:{p}_{4}$ is kept the same as y-coordinate of the point $\:{p}_{2}$. Therefore, $\:{p}_{3}=\left(\frac{1}{3},\frac{1}{3}\right)$ and $\:{p}_{4}=\left(\frac{1}{6},\frac{2}{3}\right)$. Further, a similar procedure is employed to obtain two points for the right rectangle. Precisely, $\:{p}_{6}$ will be kept the same as $\:{p}_{2}$ in the first iteration. However, the x-coordinate of the point $\:{p}_{5}$ is calculated by adding the length of right rectangle along the x-direction (direction of division in the first iteration) to the x-coordinate of the point $\:{p}_{1}$ while the y-coordinate of the point $\:{p}_{5}$ is kept the same as y-coordinate of the point $\:{p}_{1}$. Therefore, $\:{p}_{5}=\left(\frac{5}{6},\frac{1}{3}\right)$ and $\:{p}_{6}=\left(\frac{2}{3},\frac{2}{3}\right)$. Furthermore, if the right rectangle satisfies the conditions illustrated in Eqs. (3) and (4) then it will be candidate one for partitioning as shown in the second iteration in Fig. 1. In addition, the same procedure will be applied in the subsequent iterations. Consequently, in the third iteration, if the most left and the right bottom rectangles are the candidate ones for partitioning, then the right bottom rectangle will be divided along x-direction as it is a square while the most left rectangle is divided along y- direction (the longest dimension). The proposed optimization technique is outlined in the flowchart shown in Fig. 2.

### Design consideration and simulation discussions

**Fig. 3:**
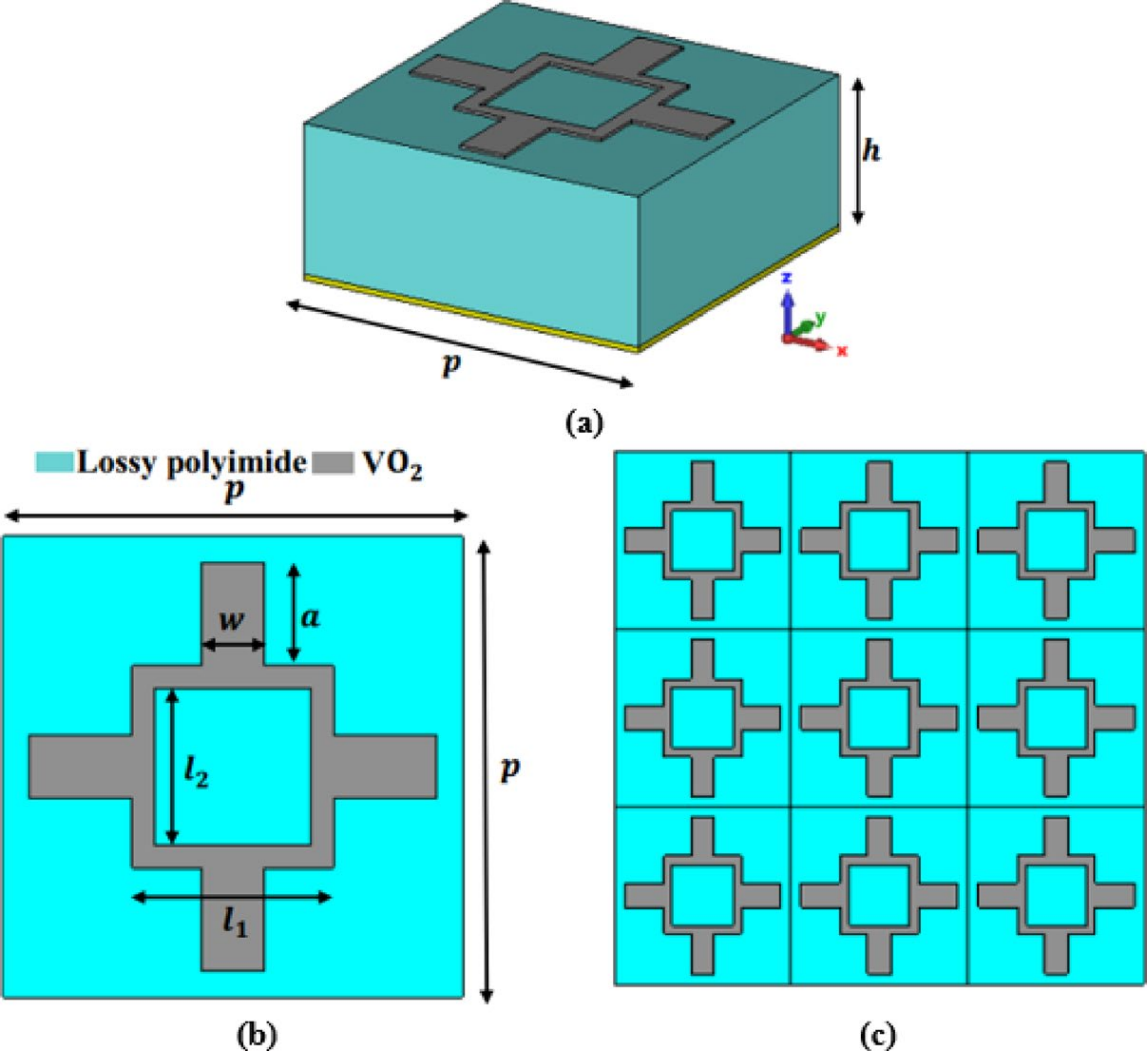
The proposed design structure; (**a**) 3D view of the proposed structure, (**b**) 2D cross-sectional of investigated absorber and (**c**) periodic structure of the studied design.

A three-dimensional view of a unit cell of the investigated design is demonstrated in Fig. 3a. The introduced structure comprises of three layers. The top layer is a vanadium dioxide (VO_2_) resonator formed by squares and cross geometries with a thickness of $\:0.2\:\mu\:$m. The VO_2_ has tunable characteristics when changing its phase from insulator to a metallic phase thermally or by applying an external electric field. A two-dimensional cross section of the design unit cell is illustrated in Fig. 3b and the associated patch array with periodicity $\:\left(p\right)$ is depicted in Fig. 3c. The bottom layer is composed of a continuous gold layer with electric conductivity $\:\sigma\:=5.8\times\:{10}^{7}$ S/m and a thickness of $\:0.4\:\mu\:$m. This layer performs as a ground metallic plane that prohibits any transmission from the proposed absorber. Further, a polyimide dielectric layer with a dielectric permittivity of $\:\epsilon\:=3.5(1+i0.0027)$^42^ and a thickness of $\:h=10\:\mu\:$m is used between the VO_2_ and the ground layers.

The simulation results of the introduced design are obtained using the frequency domain solver of the CST software package^41^. In this study, open boundary conditions are set al.ong the z- direction, while periodic boundary conditions are selected in the x- and y- directions. Further, an adaptive mesh with 240,416 tetrahedrons is employed during the simulation to achieve high accuracy. It should be noted that the conductivity of VO_2_ can be varied from 200 to $\:3\times\:{10}^{5}\:$S/m in few picoseconds under heat or the external stimulation of electric field^43^. Therefore, VO_2_ can be employed as an active material to achieve controllable optical properties. Furthermore, the conductivity of VO_2_ is presented through the following Drude model^28^

5 $$\:\epsilon\:\left(\omega\:\right)={\epsilon\:}_{{\infty\:}}-\frac{{\omega\:}_{p}^{2}\left(\sigma\:\right)}{{\omega\:}^{2}+ i\omega\ /\tau, }$$

where $\:{\epsilon\:}_{{\infty\:}}=12$ and $\:\tau\:=2.27\times\:{10}^{-12}\mathrm{s}$ are the permittivity of VO_2_ at an infinite frequency, the relaxation time, respectively. Further, the dependence of the plasma frequency on the conductivity at THz frequency can be presented as

6 $$\:{\omega\:}_{p}^{2}\left(\sigma\:\right)=\frac{\sigma\:}{{\epsilon\:}_{0}\tau\:}\:,$$

where $\:{\epsilon\:}_{0}\:$is the vacuum permittivity.

The goal of achieving the optimal absorber characteristics can be realized through maximizing the absorptivity ($\:A$) of the MMA at a specific frequency $\:f$ that can be formulated as:

7 $$\:A=1-{\left|{S}_{11}\left(f\right)\right|}^{2}-{\left|{S}_{21}\left(f\right)\right|}^{2}\:,$$

where $\:{S}_{11}\left(f\right)$ and $\:{S}_{21}\left(f\right)\:$denote the reflection and transmission coefficients, respectively. In this study, the bottom metallic layer has a sufficiently large thickness compared to the wave penetration depth through the gold layer. Hence, there is no transmission through the investigated design and the absorptivity can be evaluated as:

8 $$\:A=1-{\left|{S}_{11}\left(f\right)\right|}^{2},$$

**Fig. 4:**
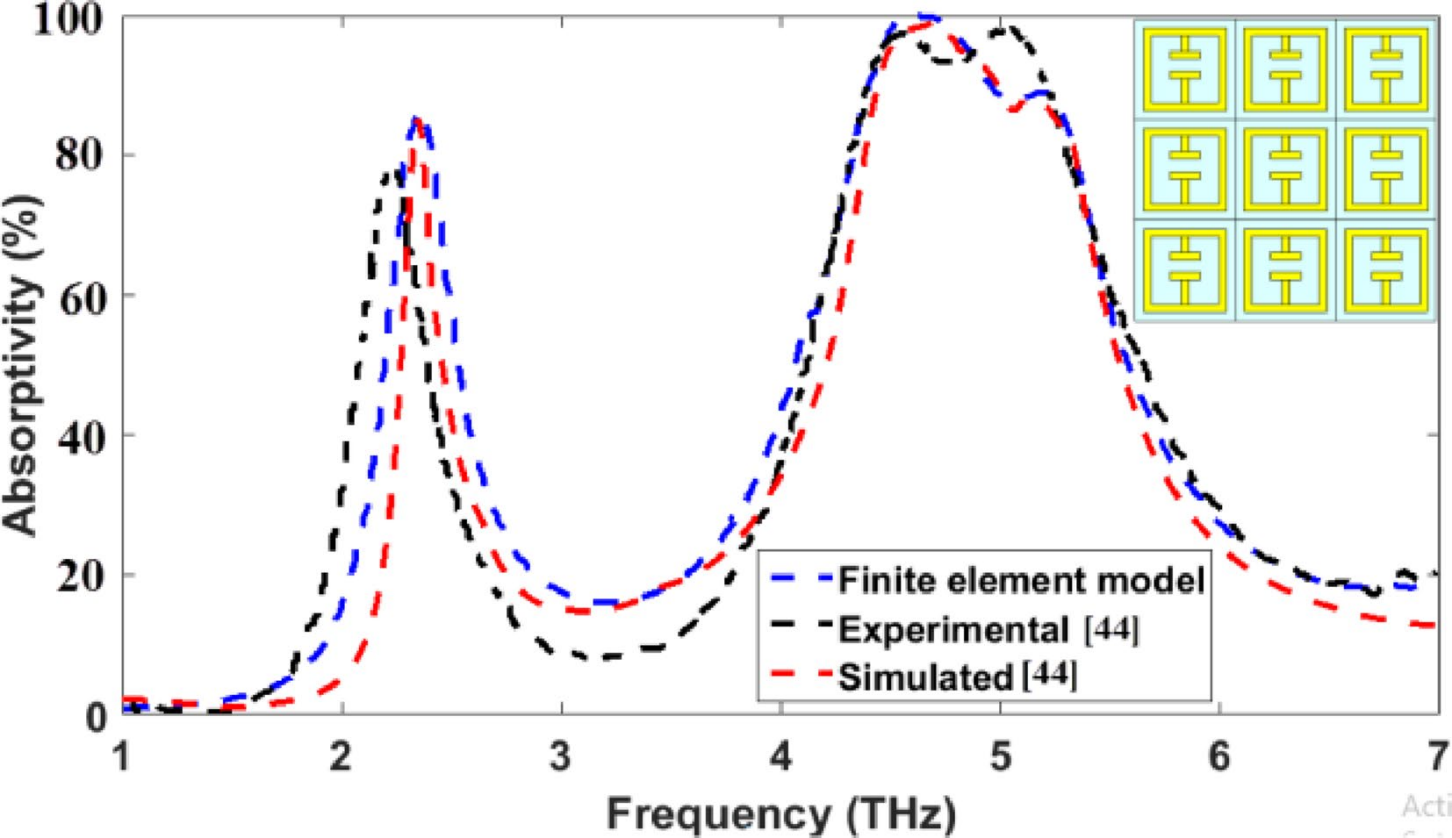
A comparative study between the employed finite element model and the simulated and measured results studied in^44^.

Initially, a comparative study will be performed between the simulated and experimental results of the MMA reported in^44^ as presented in Fig. 4 to validate the accuracy of the employed full vectorial finite element simulation model. The studied design in^44^ is illustrated in the inset of Fig. 4. The figure clearly demonstrates a strong agreement between the results obtained based on the employed simulation model and the reported results in^44^.

Subsequently, the optimization of the proposed MMA is studied using the suggested BIRECT optimization algorithm. To achieve a high absorptivity over a wide band of frequencies, the reported optimization algorithm will be employed to maximize the following objective function

9 $$\:m\left(\boldsymbol{x}\right)=\frac{\sum\:_{f=2THz}^{f=6\:THz}\:A\:\left(f\right)}{\mathrm{n}\mathrm{u}\mathrm{m}\mathrm{b}\mathrm{e}\mathrm{r}\:\mathrm{o}\mathrm{f}\:\mathrm{f}\mathrm{r}\mathrm{e}\mathrm{q}\mathrm{u}\mathrm{e}\mathrm{n}\mathrm{c}\mathrm{i}\mathrm{e}\mathrm{s}}\:\:,$$

where $\:\boldsymbol{x}=\left[p,\:{l}_{1},\:{l}_{2},\:a,\:w\right]$ is the vector of design parameters.

During the optimization iterations, 4000 frequency samples are considered along the investigated frequency band from 2 THz to 6 THz with a step of 1 GHz.

The steps of the proposed optimization algorithm to achieve the optimal design are as follows:

1. $\:\left(n+1\right)\left(n+2\right)/2$ sample points will be generated around the initial candidate point $\:{\boldsymbol{x}}_{0}$ using the Latin hypercube sampling. In this paper, $\:n=5$ is the number of design parameters. Therefore, 21 points will be generated and the objective function $\:m\left(\boldsymbol{x}\right)$ is calculated at each point using the finite element model (CST solver).
2. At each iteration, $\:k$, a quadratic surrogate model with $\:\left(n+1\right)\left(n+2\right)/2$ unknowns will be generated. This quadratic model takes the form $\:s\left(\boldsymbol{x}\right)=a+{\boldsymbol{b}}^{T}\boldsymbol{x}+\frac{1}{2}\:{\boldsymbol{x}}^{\boldsymbol{T}}\boldsymbol{H}\boldsymbol{x}$, where $\:a\in\:\mathbb{R}$, $\:\boldsymbol{b}\in\:{\mathbb{R}}^{n}$, $\:\boldsymbol{H}\in\:{\mathbb{R}}^{n\times\:n}\:$is a symmetric matrix. This model will be generated by solving $\:\left(n+1\right)\left(n+2\right)/2$ equations in $\:\left(n+1\right)\left(n+2\right)/2$ unknowns using the least squares method.
3. The constructed model $\:s\left(\boldsymbol{x}\right)$ is then maximized instead of the high-fidelity objective function $\:m\left(\boldsymbol{x}\right)$ using the proposed optimization algorithm (BIRECT) to reduce the simulation time and as a result a new local optimal point is generated $\:{\boldsymbol{x}}_{k}$, then the computationally expensive objective function $\:m\left(\boldsymbol{x}\right)$ will be calculated only at this point using the finite element model (CST solver) and this point will be added to the set of available points (with a number equals $\:J)$.
4. A new quadratic model is generated by updating the quadratic model parameters ($\:a$, $\:\boldsymbol{b}$, $\:\boldsymbol{H}$) through solving $\:J$ equations in $\:\left(n+1\right)\left(n+2\right)/2$ unknowns using the least squares method and then go to step 3.
5. The proposed algorithm will be stopped when the difference between two successive objective function values is very small (less than 0.01).

**Fig. 5:**
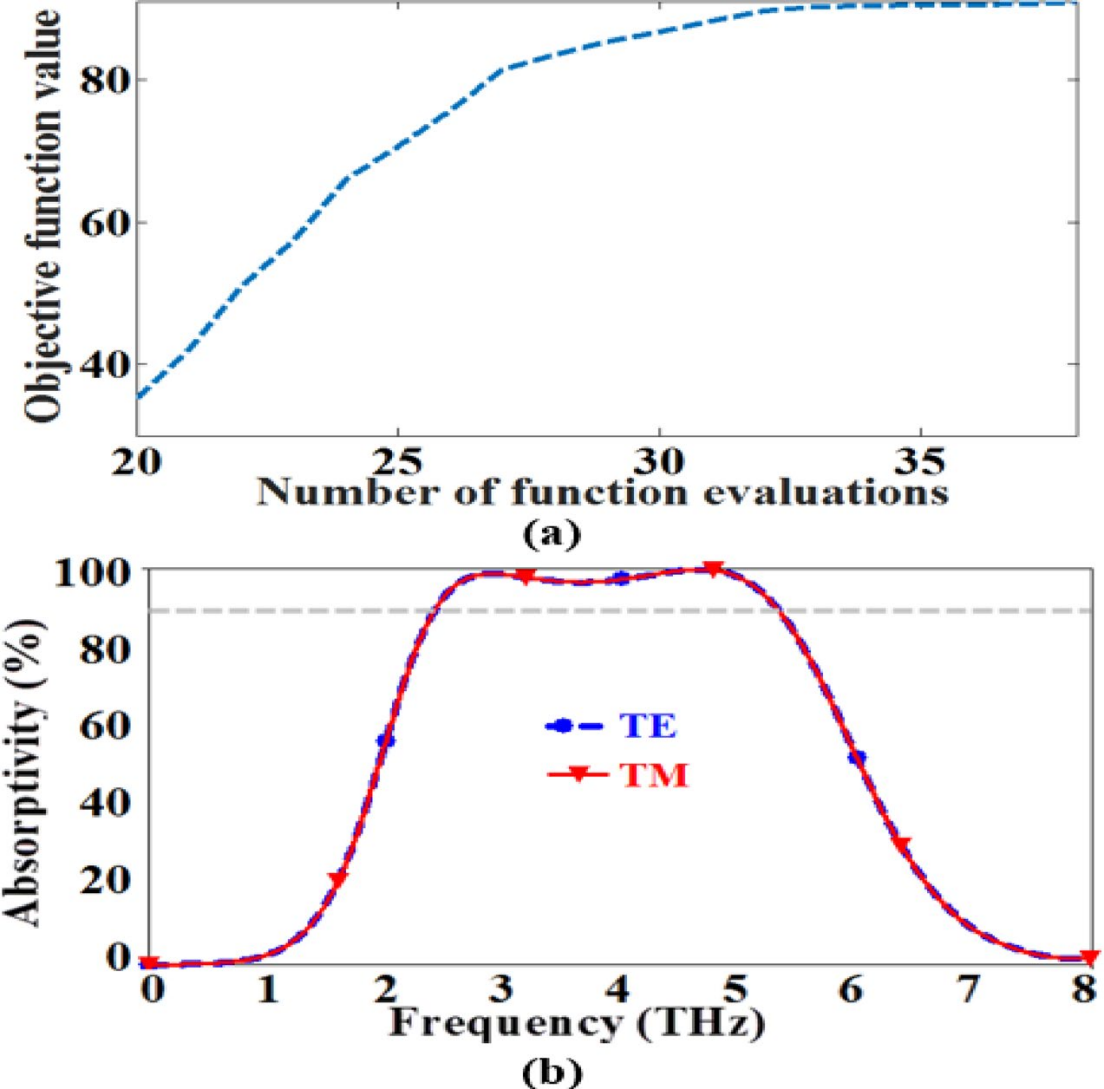
(**a**) The computationally expensive objective function values versus the computed number of the function values and (**b**) The absorption performance of the suggested absorber at normal incidence for the two polarized waves.

It is also important to note that the time consumed in evaluating the high-fidelity objective function values represents the most of the simulation time during the optimization process. Therefore, the introduced optimization technique performance is determined by calculating the required number of costly objective function computations to achieve the proposed design. The variation of the objective function values at distinct number of function computations is illustrated in Fig. 5a. It is evident from the figure that the presented optimization technique could reach the optimal design parameters after evaluating 38 objective function values with overall simulation duration of 15 h on personal PC (processor core i7, 2.7 GHz, 8 GB RAM). Table 1 shows the values of the initial and final design parameters. Further, the absorption spectra at the optimal point of the reported absorber for TE and TM polarizations at normal incidence are illustrated in Fig. 5b. The figure shows that the proposed MMA could achieve a wide bandwidth of 2.9 THz with high absorptivity (greater than 90%) between the frequencies 2.43 and 5.33 THz when the conductivity of the top VO_2_ layer is 300,000 S/m. It should be noticed that the absorption spectra of the investigated absorber coincide for the two polarization waves. Therefore, the reported THz MMA can be employed in different fields such as communication^45^, radar^46^, and stealth technology^47^.

**Table 1:** The initial and optimum values of the geometrical parameters of the reported MMA.

Geometric parameters ($\:\boldsymbol{\mu\:}\boldsymbol{m})$	Initial values ($\:\boldsymbol{\mu\:}\boldsymbol{m})$	Optimal values ($\:\boldsymbol{\mu\:}\boldsymbol{m})$
$\:p$	20	24
$\:{l}_{1}$	6	10.537
$\:{l}_{2}$	5	8.214
$\:a$	2	5.954
$\:w$	1	3.247
$\:\frac{\sum\:_{f=2}^{f=6}A\:\left(f\right)}{\mathrm{number\:of\:frequencies}}$	35.01	90.86
Bandwidth (THz)	$\:-$	$\:2.43-5.33$

To explain the absorption enhancement of the proposed MMA, the effective permittivity ($\:{\epsilon\:}_{eff})$, effective permeability ($\:{\mu\:}_{eff})$, and the effective surface impedance ($\:Z)$ of the suggested design are analyzed and calculated as follows^48^

10 $$\:{\epsilon\:}_{eff}=1+\frac{2j}{{K}_{0}h}\left(\frac{1-{S}_{11}}{1+{S}_{11}}\right)$$

11 $$\:{\mu\:}_{eff}=1+\frac{2j}{{K}_{0}h}\left(\frac{1+{S}_{11}}{1-{S}_{11}}\right)$$

12 $$\:Z=\frac{1+{S}_{11}}{1-{S}_{11}}$$

where $\:{K}_{0}$ is the wave number in free space and $\:{S}_{11}\:$is the reflection coefficient of the suggested MMA. However, the transmission of the reported design turns zero due to the continuous bottom metallic layer. Therefore, the effective material parameters will depend only on the reflection coefficient $\:{S}_{11}$ of the MMA.

**Fig. 6:**
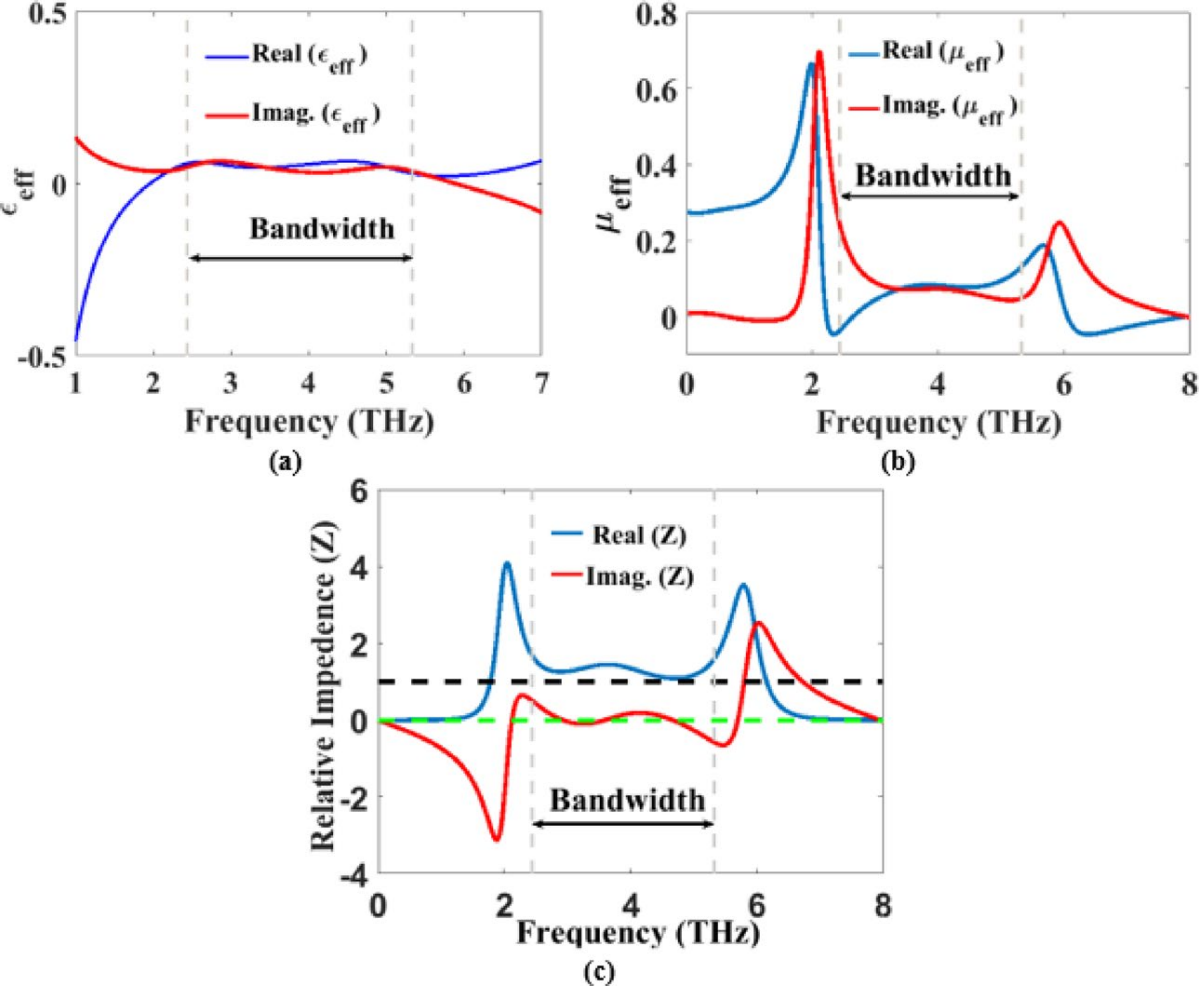
Effective material parameters of the proposed MMA; (**a**) $\:{\epsilon\:}_{eff}$ (**b**) $\:{\mu\:}_{eff}$ (**c**) $\:Z$.

The variations of the real and the imaginary parts of $\:{\epsilon\:}_{eff}$ and $\:{\mu\:}_{eff}$ are presented, respectively, in Fig. 6a, b. The figure shows that the magnitude of the real and imaginary parts of $\:{\epsilon\:}_{eff}$ and $\:{\mu\:}_{eff}$ are nearly equal at the resonance frequencies between 2.43 and 5.33 THz. As a result, the perfect absorption characteristics of the proposed absorber is obtained as reported in the following formula^49^

13 $${\mathrm{A}}({\mathrm{f}})=1 - {\left| {\frac{{\sqrt {{\mu _{{\mathrm{eff}}}}} - \sqrt {{\varepsilon _{{\mathrm{eff}}}}} }}{{\sqrt {{\mu _{{\mathrm{eff}}}}} +\sqrt {{\varepsilon _{{\mathrm{eff}}}}} }}} \right|^2}$$

Figure 6c represents the real and imaginary components of the effective surface impedance for the studied MMA. It is evident from this figure that the real part of $\:Z$ is close to unity in the investigated frequency band from 2.43 to 5.33 THz, while the imaginary part is almost zero. Therefore, a perfect matching will occur between the surface impedance of the top resonator of the reported design and the impedance in free space ($\:{Z}_{0}$). This leads to a minimum reflection and a high absorption rate from the structure of the suggested MMA in the studied bandwidth as illustrated in the following formula

14 $$\:A\:=1-{\left|\frac{Z\:-{Z}_{0}}{Z+{Z}_{0}}\right|}^{2}$$

**Fig. 7:**
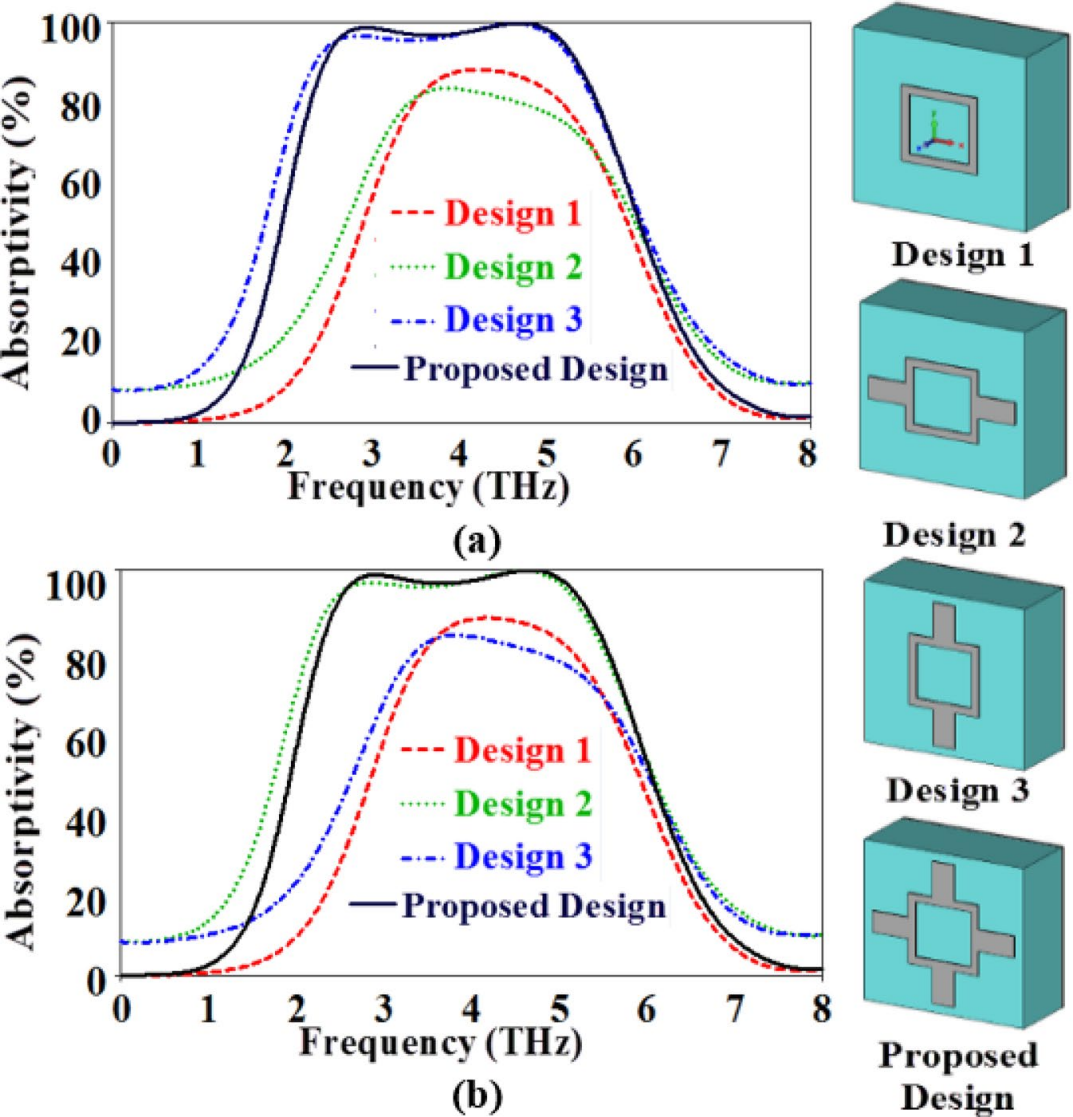
The absorption performance of the studied designs for the (**a**) TE (**b**) TM polarized waves.

The effect of the different elements in the top resonator on the performance of the suggested absorber is also studied for the TE and TM polarizations as illustrated in Fig. 7. Initially, only the two square resonators are analyzed (design 1). In this case, a narrow bandwidth with a maximum absorptivity less than 90% is achieved for the two polarized waves. Next, in design 2, two horizontal arms are added which leads to increasing the bandwidth to be 2.95 THz between the frequencies 2.33 and 5.28 THz with high absorptivity (above 90%) for only the TM polarized wave, while the maximum absorptivity is below 90% with a narrow bandwidth for the TE polarized wave. However, adding two vertical arms as illustrated in design 3 in the inset of Fig. 7 leads to exchanging the absorption spectra of the two polarization waves that are obtained in design 2. Therefore, the vertical and horizontal arms are combined in the introduced absorber to have a symmetric structure with a perfect absorption performance in a wide THz range of frequencies between 2.43 and 5.33 THz for the two polarization waves.

**Fig. 8:**
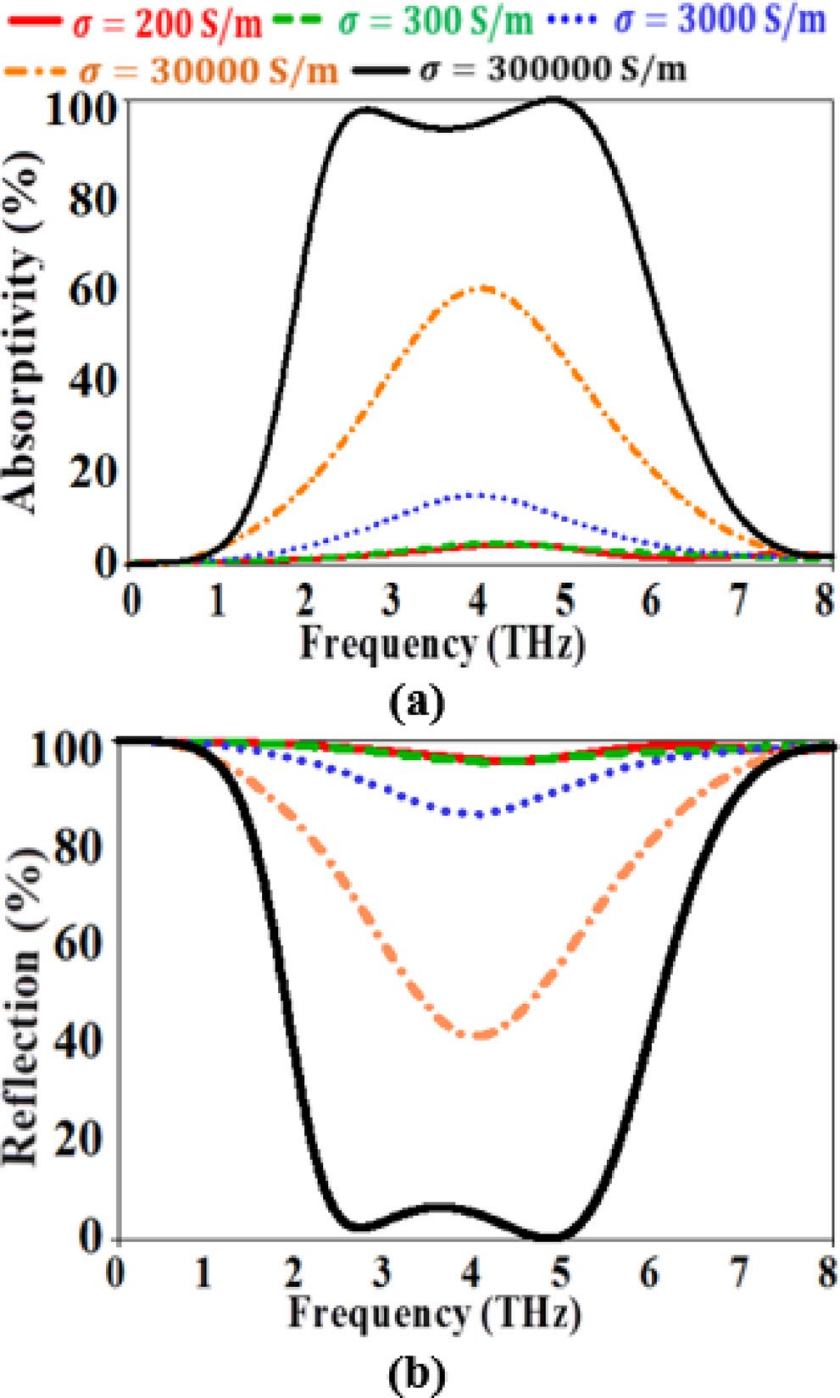
The variations of the (**a**) absorption spectrum (**b**) Reflection spectrum with different electrical conductivities of VO_2_.

To clarify the tunable performance of the proposed MMA, the impact of varying the conductivity of the VO_2_ material on the absorption and reflection spectra is studied as illustrated in Fig. 8. It is evident from the figure that increasing the conductivity from 200 S/m to 300,000 S/m leads to increasing the maximum absorptivity from 5% to be 99.99% in the studied frequency range. However, the reflection is reduced by increasing the conductivity of the VO_2_ material as shown in Fig. 8b. Therefore, the introduced design is a tunable bi-functional metamaterial device that can be used as a broadband absorber or a broadband reflector depending on the conductivity of the top layer resonator.

**Fig. 9:**
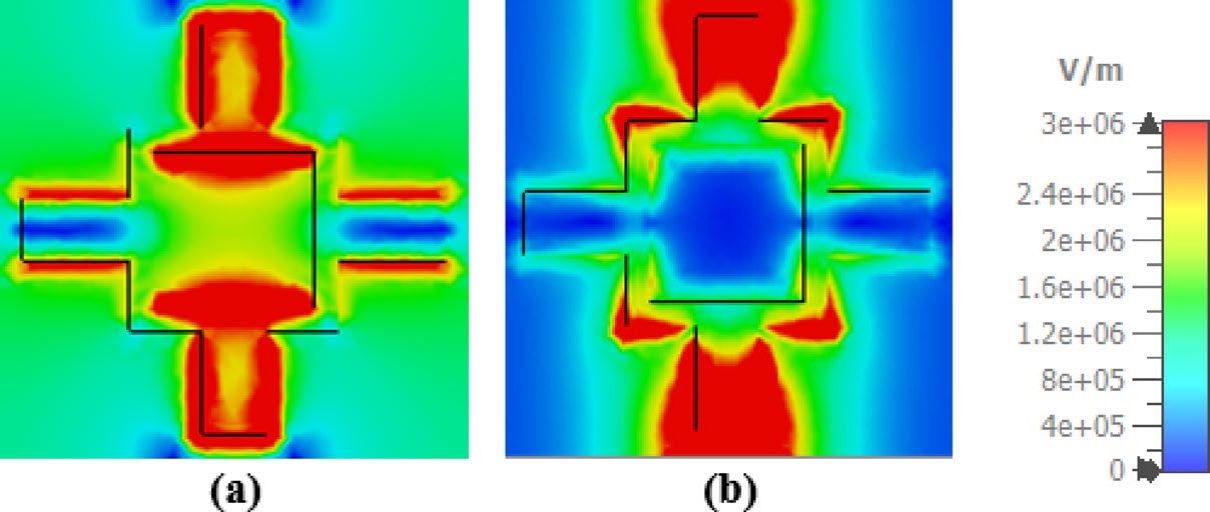
Electric field distribution of the TE polarization of the reported absorber at resonance frequencies of (**a**) 2.91 THz, and (**b**) 4.67 THz.

**Fig. 10:**
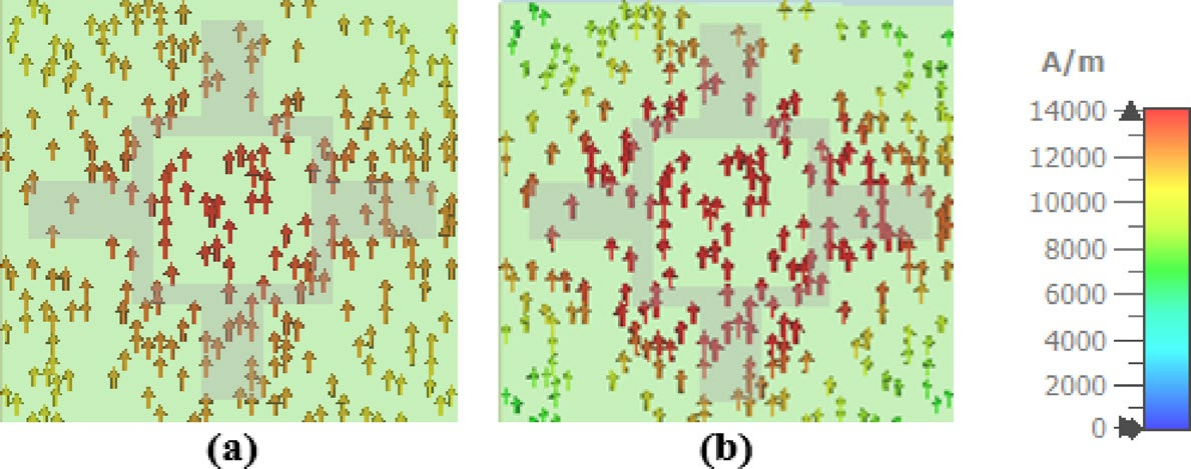
Surface current density of the reported absorber at resonance frequencies of (**a**) 2.91 THz, and (**b**) 4.67 THz.

The absorption enhancement of the introduced THz absorber is also clarified by analyzing the normalized electric field distributions and the surface current density for the TE polarization wave at the resonance frequencies of 2.91 THz, 4.67 THz as shown in Figs. 9 and 10 respectively. The results illustrated in Fig. 9 show that the electric field distributions are conspicuous at the arms and the corner edges of the VO_2_ resonator. As a result, an electric resonance is excited at the top resonator inducing an opposite electric resonance in the gold layer. Accordingly, surface currents are produced on the resonator as illustrated in Fig. 10 causing a magnetic resonance. The occurrence of simultaneous electric and magnetic resonances results in obtaining a high absorptivity in a wide range of frequencies^50^.

**Fig. 11:**
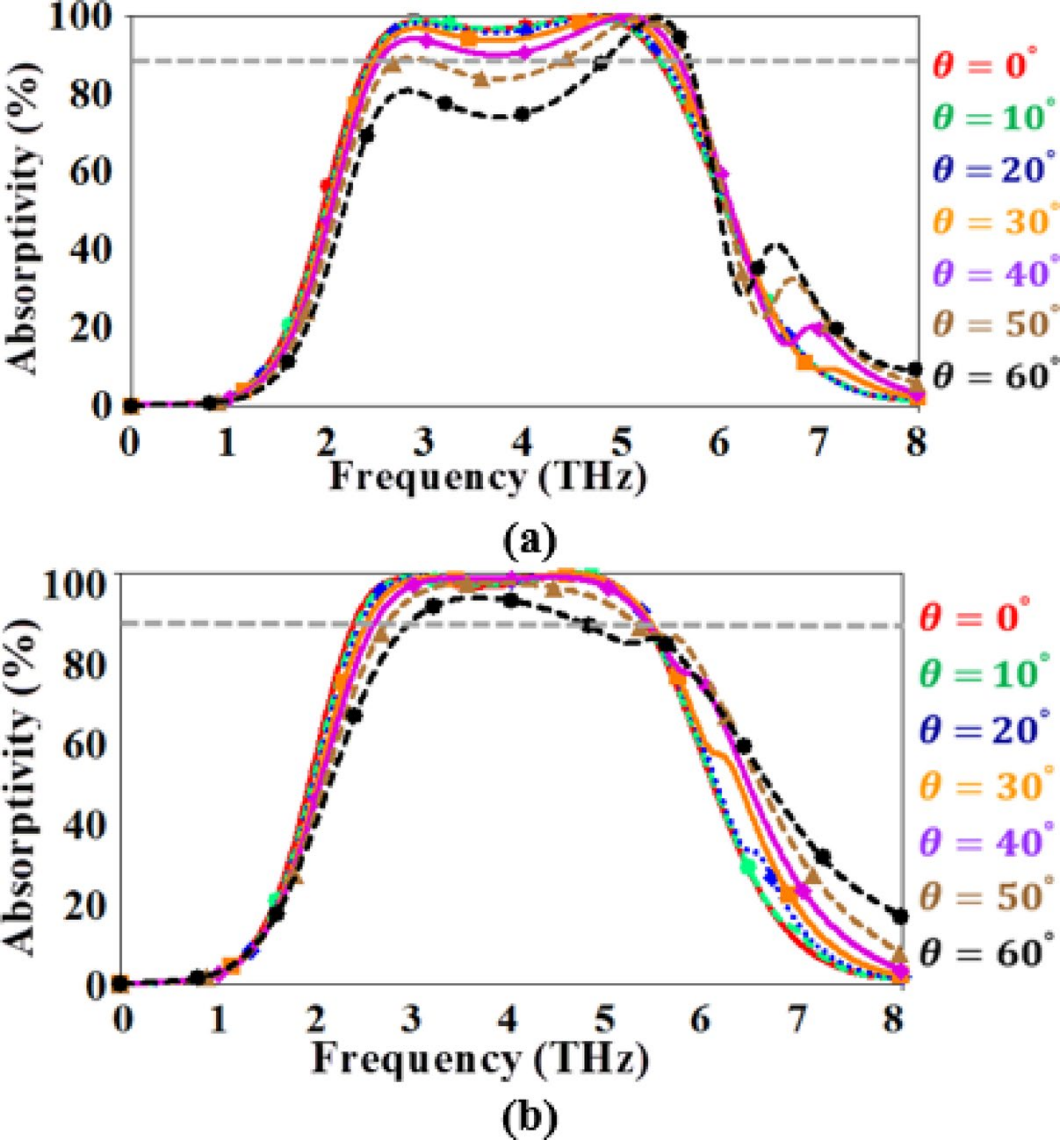
The absorption spectrum of the reported MMA over various angles of the incident wave for (**a**) TE mode and (**b**) TM mode.

Figure 11 exhibits the variation of the absorption spectrum with the angle of the incident wave $\:\left(\theta\:\right)$ for the two polarization waves. It is worth noting that increasing the incident angle leads to decreasing the absorptivity. In addition, the reported THz absorber could obtain a robust absorption performance with a high absorptivity (above 80%) over a wide angular range (up to $\:{50}^{^\circ\:}$) for the two polarization waves.

**Fig. 12:**
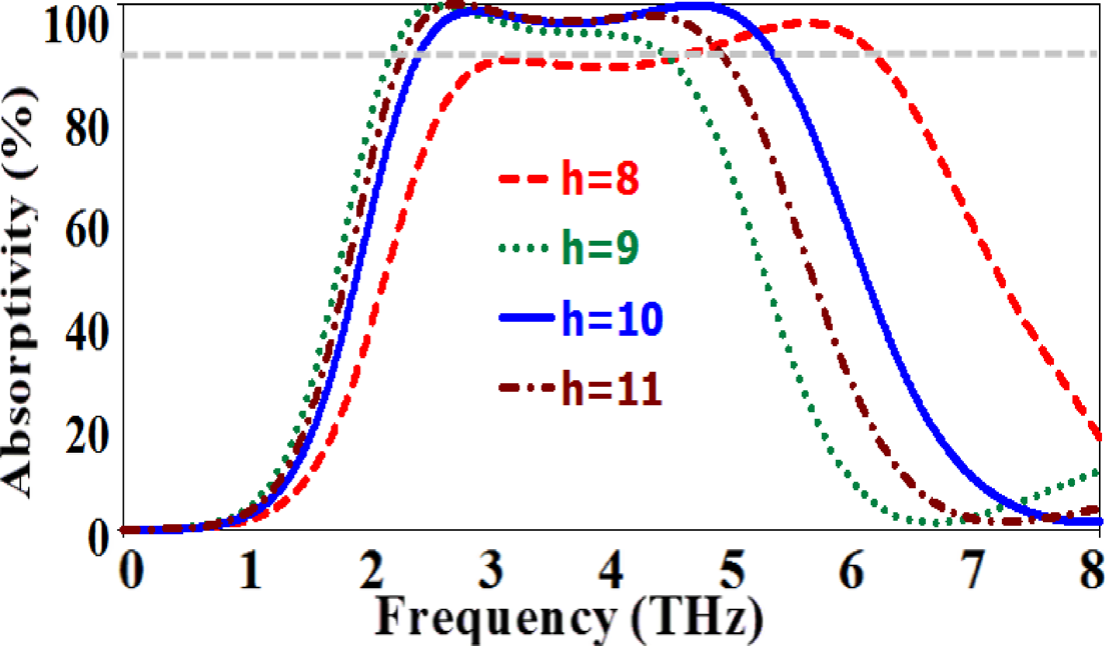
The absorption response of the suggested MMA at various values of dielectric substrate thickness.

Figure 12 illustrates the variation of the absorption response of the presented absorber with the dielectric substrate thickness $\:\left(h\right)$. The figure shows clearly that the best absorption response could be realized when the dielectric substrate thickness equals 10 $\:\mu\:$m.

The fabrication of the introduced geometrical structure can be efficiently achieved through the photolithography technique^51^. During the manufacturing process, the middle substrate layer is made of polyimide which is usually deposited via spin coating of a liquid precursor, and then a high temperature curing process is employed to solidify the film and achieve the desired dielectric properties. The molecular beam epitaxy technique is employed to coat this layer from up with a uniform layer of the VO_2_ material. However, the electron beam lithography technique is employed to deposit a gold layer on the bottom of the polyimide substrate. Subsequently, a photoresist layer is used to coat the uniform layer of the VO_2_ material and a clear photolithography mask containing the desired resonator pattern (a square with horizontal and vertical arms as suggested in the proposed resonator structure) is precisely aligned and placed above the photoresist layer. Further, an ultraviolet light source is incident to the mask, exposing the photoresist beneath the transparent sections. Moreover, a reactive ion etching process with an appropriate gas chemistry is employed to selectively remove the unprotected VO_2_​ down to the polyimide layer leaving behind only the VO_2_​ trace pattern covered by the remaining photoresist which is stripped off using a solvent.

It is worth noting that there are several works in the literature that have used the photolithography process in the fabrication of THz and GHz metamaterial absorbers employing VO_2_ resonators or other types of resonators. For example, the fabrication of a VO_2_ based THz nano-antennas using the photolithography technique is described in^52^. Further, the patterning of the VO_2_ layer using photolithography process is illustrated in^53^ to obtain THz metamaterial filters. In addition, the work provided in^54^ detailed a potential fabrication process that included multiple steps of photoresist spin-coating, photolithography, and lift-off processes to pattern gold and VO_2_ layers. Therefore, the photolithography technique is widely used in the literature in the fabrication of THz devices employing VO_2_ resonators. The phase transition temperature of the VO_2_​ material $\:({68}^{^\circ\:}C$ or approximately 340 K) can be achieved through electrical heating, specifically Joule heating, by applying a controlled voltage or current across integrated electrodes. This method allows for rapid, localized heating that dramatically alters its electrical conductivity and thus tuning the metamaterial’s THz absorption properties. It has been experimentally demonstrated that applying a DC bias across the integrated electrodes successfully triggers the VO_2_​ material phase transition^55^, leading to a dynamic change in the THz response. It should be noticed that the operation of inducing a phase transition in the VO_2_​ material using integrated electrodes via Joule heating is considered stable and highly reliable for practical engineering applications. Experimental studies have specifically tested the endurance of these VO_2_​ based devices over millions of operational cycles to confirm their stability^56^. Further, the time required for the VO_2_​ phase transition using integrated electrodes can range from milliseconds (ms) down to sub-nanoseconds (ns)^57^.

The proposed design is suitable for integration into the existing systems in different applications. For example, the use of a flexible polyimide substrate is a primary advantage for integration onto curved surfaces like aircraft bodies, so it can be used for stealth applications^58^. Further, the absorber can be integrated into the signal pathway of a transceiver module as a rapidly switchable filter or modulator that can dynamically tune absorption bands to manage specific frequency channels, acting as a reconfigurable front-end component in 6G base stations^59^. Furthermore, the ability to dynamically switch the absorption properties makes the proposed design valuable for advanced imaging and biochemical sensing systems^60^.

Also, a tolerance investigation for all design parameters is performed as shown in Table 2 to prove that the performance of the presented design is immune against the fabrication defects. In this investigation, only single design parameter is varied at a time by $\:\pm\:5\mathrm{\%}$ from its optimal value. Table 2 shows that the proposed absorber could keep the maximum absorptivity above 99% with a bandwidth greater than 2.68 THz in all cases.

It is also worth noting that the employed materials (gold, polyimide, vanadium dioxide (VO_2_)) are widely used for metamaterial absorbers in the literature^27,29,31,33,34^. Further, the gold material is only used as a ground layer to prevent any transmissions with a sufficient thickness. Also, the polyimide dielectric layer is widely used with stable electrical properties in the THz range, allowing for predictable and reliable absorber performance with high absorption over a wide range of incident angles, making these absorbers practical for real-world applications^61^. Moreover, the use of fabricated VO_2_ resonators with reliable and stable operational characteristics is standard in the field of tunable THz metamaterials. For example, Jiao et al.^62^ discussed how embedding the VO_2_ resonator prevents degradation and ensures stable performance. Also, Zhou et al.^63^ highlighted the excellent angular stability and polarization-insensitive characteristics of a VO_2_ resonator. In addition, Hasan et al.^64^ noted that using a VO_2_ resonator provides stable absorption characteristics over a broad span of incident angles. Based on the mentioned material stability, it is believed that the proposed design has the advantages of stable material characteristics, which offer high robust performance.

**Table 2:** A variational analysis for the proposed THz absorber.

Geometrical parameters	Perturbation (%)	Supreme value of absorptivity (%)	Frequency range (THz)
$\:p$	$\:+5$	99.83	2.47–5.28
	$\:-5$	99.64	2.45–5.25
$\:{l}_{1}$	$\:+5$	99.34	2.48–5.24
	$\:-5$	99.89	2.51–5.19
$\:{l}_{2}$	$\:+5$	99.86	2.48–5.17
	$\:-5$	99.67	2.47–5.28
$\:a$	$\:+5$	99.77	2.42–5.31
	$\:-5$	99.76	2.46–5.23
$\:w$	$\:+5$	99.75	2.45–5.21
	$\:-5$	99.76	2.44–5.33

Table 3 shows a comparative study between the suggested absorber and some wideband THz absorbers in the literature^26–34^ to show the prevalence and strength of the reported design. It is revealed from Table 3 that the introduced MMA has a wider absorption bandwidth compared to most of the previously reported THz absorbers. It may be noticed that the THz MMAs suggested in^27,29^ have a wider absorption bandwidth than the suggested design. However, the THz MMAs introduced in^27,29^ have larger dielectric substrate thickness, unit cell size and number of layers compared to the investigated design. Subsequently, the proposed THz absorber has several traits such as compact unit cell size, ultrathin thickness, polarization insensitive, broad bandwidth and robust absorption response over a wide angular range. These advantages make the reported design a strong candidate in communication, stealth technology and other different applications.

**Table 3:** Comparison of the presented THz absorber with some reported studies in the literature.

Shape/resonator material	Absorption band (THz)	Periodicity ($\:\mu\:\mathrm{m}$)	Thickness ($\:\mu\:\mathrm{m}$)	Supreme value of the absorptivity (%)	Number of layers
This work	2.9 (2.43–5.33)	24	10	99.99	3
Four triangular graphene resonators^26^	1.96 (3.287–5.247)	5	14.7	99.12	3
Split ring-shaped graphene MMA^27^	2.18 (2.06–4.24) 3.14(1.33–4.47)	23 27	15 30	> 90 > 90	3 4
Asymmetric square Split-Ring array^28^	0.65 (0.98–1.63)	15	28	99.3	3
Four L-shaped VO_2_ patches MMA^29^	1.87 (1.31–3.18) 3.95 (4.45–8.4)	25	21.5	> 90	6
Disk structure surrounded by a square ribbon^30^	2.173 (1.482–3.655)	40	21	> 90	3
Hybrid metal-graphene-based MMA^31^	1 (0.95–1.95)	15	28	98.9	3
Four layers of graphene squares^32^	2.9 (1.45–4.35)	30	22	> 90	4
Six layers MMA based on VO_2_ resonator^33^	2.29 (3.33–5.62)	10	8.84	99	6
Four layers MMA based on graphene and VO_2_^34^	2.24 (5.8–8.04)	10	13.4	> 90	5

## Conclusion

In this article, a global optimization technique that bisects the design space into smaller hyperrectangles is integrated with a quadratic surrogate model and full vectorial finite element method to obtain the optimal design of an ultrathin broadband THz MMA. The proposed design comprises of VO_2_ resonators that are designed with squares and cross geometries over a dielectric polyimide layer. The suggested absorber could achieve a wide absorption bandwidth of 2.9 THz with high absorptivity exceeding 90% between the frequencies 2.43 THz and 5.33 THz. Further, the variation of the absorption performance over various angles of the incident wave is studied and the reported design could maintain the absorptivity above 80% for a wide angular range (up to 50°). In addition, the introduced absorber is polarization insensitive with a robust behavior against the fabrication imperfection. Subsequently, the reported absorber can be employed in several THz applications such as radar and stealth technology. Furthermore, the suggested optimization technique could verify powerful and strength for achieving the optimal geometrical parameters of broadband THZ MMAs.

## Data Availability

The datasets used and/or analyzed during the current study available from the corresponding author on reasonable request.

## References

[1] Smith, D. R., Padilla, W. J., Vier, D. C., Nemat-Nasser, S. C. & Schultz, S. Composite medium with simultaneously negative permeability and permittivity. *Phys. Rev. Lett.* **84**, 4184–4187 (2000). 10990641 10.1103/PhysRevLett.84.4184

[2] Schurig, D. et al. Metamaterial electromagnetic cloak at microwave frequencies. *Science* **314**, 5801, 977–980 (2006). 17053110 10.1126/science.1133628

[3] Pendry, J. B. Negative refraction makes a perfect lens. *Phys. Rev. Lett.* **85** (18), 3966 (2000). 11041972 10.1103/PhysRevLett.85.3966

[4] Xiao, Z. Y., Liu, D. J., Ma, X. L. & Wang, Z. H. Multi-band transmissions of chiral metamaterial based on Fabry–Perot like resonators. *Opt. Express*. **23** (6), 7053–7061 (2015). 25837050 10.1364/OE.23.007053

[5] Javed, I. et al. Broad-band polarization-insensitive metasurface holography with a single-phase map. *ACS Appl. Mater. Interfaces*. **14** (31), 36019–36026 (2022). 35912417 10.1021/acsami.2c07960

[6] Zhou, Z. et al. High performance metamaterials-high electron mobility transistors integrated terahertz modulator. *Opt. Exp.* **25**, 17832–17840 (2017). 10.1364/OE.25.017832 28789274

[7] Dai, L., Zhang, Y., Zhang, H. & O’Hara, J. F. Broadband tunable terahertz cross-polarization converter based on Dirac semimetals. *Appl. Phys. Express*. **12**, 7, 075003 (2019).

[8] Etman, A. S., Hameed, M. F. O., Obayya, S. S. & Hammad, A. E. Optimization and fabrication of highly sensitive metamaterial sensor using dividing rectangles-based kriging surrogate model. *IEEE Sens. J.* **24** (6), 8054–8063 (2024).

[9] Jornet, J. M. & Akyildiz, I. F. Graphene-based plasmonic nanoantenna for terahertz band communication in nanonetworks. *IEEE J. Sel. Areas Commun.* **31** (12), 685–694 (2013).

[10] Lu, F., Ou, H. & Lin, Y. S. Reconfigurable terahertz switch using flexible L-shaped metamaterial. *Opt. Lett.* **45**, 23, 6482–6485 (2020). 33258842 10.1364/OL.402949

[11] Hengbo, X. Design, simulation, and measurement of a multiband tunable metamaterial filter. *Opt. Mater.* **127**, 112253 (2022).

[12] Xiong, H. et al. A metamaterial energy power detector based on electromagnetic energy harvesting technology. *ACS Appl. Electron. Mater.* **6** (2), 1204–1210 (2024).

[13] Jepsen, P. U., Cooke, D. G. & Koch, M. Terahertz spectroscopy and imaging – modern techniques and applications. *Laser Photonics Rev.* **5**, 124–166 (2011).

[14] Bilal, R., Naveed, M., Baqir, M., Ali, M. & Rahim, A. Design of a wideband terahertz metamaterial absorber based on pythagorean-tree fractal geometry. *Opt. Mater. Exp.* **10** (12), 3007–3020 (2020).

[15] Akyildiz, I. F., Jornet, J. M. & Han, C. Terahertz band: next frontier for wireless communications. *Phys. Commun.* **12**, 16–32 (2014).

[16] Dragoman, D. & Dragoman, M. Terahertz fields and applications. *Prog. Quantum Electron.* **28**(1), 1–66 (2004).

[17] Du, X. et al. A polarization-and angle-insensitive broadband tunable metamaterial absorber using patterned graphene resonators in the terahertz band. *Opt. Laser Technol.* **132**, 106513 (2020).

[18] Zakir, S. et al. Polarization-insensitive, broadband, and tunable terahertz absorber using slotted-square graphene meta-rings. *IEEE Photonics J.* **15** (1), 1–8 (2022).

[19] Dai, W. et al. Synthesis of yolk- shell structured carbonyl iron@void@nitrogen doped carbon for enhanced microwave absorption performance. *J. Alloy Compd.* 812 (2020).

[20] Xiong, Y. et al. Synergistic effect of silica coated porous rodlike nickel ferrite and multiwalled carbon nanotube with improved electromagnetic wave absorption performance. *J. Alloy Compd.* **802**, 364–372 (2019).

[21] Landy, N. I., Sajuyigbe, S., Mock, J. J., Smith, D. R. & Padilla, W. J. Perfect metamaterial absorber. *Phys. Rev. Lett.* **100**(20), 207402 (2008). 18518577 10.1103/PhysRevLett.100.207402

[22] Tao, H. et al. Highly flexible wide angle of incidence terahertz metamaterial absorber: Design, fabrication, and characterization. *Phys. Rev. B*. **78**(24), 241103 (2008).

[23] Wang, X., Wang, J., Hu, Z. D., Liu, G. & Feng, Y. Angle insensitive broadband terahertz wave absorption based on molybdenum disulfide metamaterials. *Superlattices Microstruct.* **135**, 106246 (2019).

[24] Kenney, M. et al. Octave-spanning broadband absorption of terahertz light using metasurface fractal-cross absorbers. *ACS Photon*. **4** (10), 2604–2612 (2017).

[25] Ding, F., Cui, Y., Ge, X., Jin, Y. & He, S. Ultra-broadband microwave metamaterial absorber. *Appl. Phys. Lett.* **100** (10), 103506 (2012).

[26] Zhang, K., Dong, S., Wu, X., Yu, K. & Liu, Y. Graphene-based tunable broadband metamaterial absorber for terahertz waves. *Opt. Laser Technol.* **180**, 111490 (2025).

[27] Ri, K. J., Kang, R. J. & Ri, C. H. Tunable ultra-broadband Terahertz metamaterial absorbers based on complementary split ring-shaped graphene. *AIP Adv.* **14**(5) (2024).

[28] Zhou, R. et al. Tunable broadband terahertz absorber based on graphene metamaterials and VO2. *Opt. Mater.* **114**, 110915 (2021).

[29] Wu, B. et al. Bi-channel switchable broadband terahertz metamaterial absorber. *IEEE Photonics Technol. Lett.* **35** (1), 15–18 (2022).

[30] Norouzi-Razani, A. & Rezaei Broadband polarization insensitive and tunable terahertz metamaterial perfect absorber based on the graphene disk and square ribbon. *Micro Nanostruct.* **163**, 107153 (2022).

[31] Sabaruddin, N. R. et al. Designing a broadband terahertz metamaterial absorber through bi-layer hybridization of metal and graphene. *Plasmonics* **19** (6), 3259–3272 (2024).

[32] Liu, W., Song, Z. & Wang, W. A high-performance broadband terahertz absorber based on multilayer graphene squares. *Phys. Scr.* **96** (5), 055504 (2021).

[33] Niu, J., Yao, Q., Mo, W., Li, C. & Zhu, A. Switchable bi-functional metamaterial based on vanadium dioxide for broadband absorption and broadband polarization in terahertz band. *Opt. Commun.* **527**, 128953 (2023).

[34] Wang, F. et al. Narrow-broadband switchable THz absorber based on graphene and VO2. *Opt. Express*. **33** (13), 28627–28639 (2025). 40798376 10.1364/OE.563708

[35] Najafi, A., Soltani, M., Chaharmahali, I. & Biabanifard, S. Reliable design of THz absorbers based on graphene patterns: exploiting genetic algorithm. *Optik* **203**, 163924 (2020).

[36] Bordbar, A., Basiry, R. & Yahaghi, A. Design and equivalent circuit model extraction of a broadband graphene metasurface absorber based on a hexagonal spider web structure in the terahertz band. *Appl. Opt.* **59** (7), 2165–2172 (2020). 32225748 10.1364/AO.385476

[37] Viana, A., Sousa, J. P. & Matos, M. A. Constraint oriented neighborhoods – a new search strategy in metaheuristics. In *Progress as Real Problem Solvers* 389–414 (2005).

[38] Freitas, F. G., Maia, C. L. B., Campos, G. A. L. & Souza, J. T. Optimization in software testing Using metaheuristics. *Revista de Sistemas de Informação da FSMA*. **5**, 3–13 (2010).

[39] Paulavičius, R., Chiter, L. & Žilinskas, J. Global optimization based on bisection of rectangles, function values at diagonals, and a set of Lipschitz constants. *J. Global Optim.* **71**, 5–20 (2018).

[40] Powell, M. J. The NEWUOA software for unconstrained optimization without derivatives. In *Large-Scale Nonlinear Optimization* 255–297 (2006).

[41] Studio, C. S. T. & Microwave CST Microwave studio. CST Studio Suite http://www.cst.com (2008).

[42] Li, Y. et al. Tunable broadband metamaterial absorber with single-layered graphene arrays of rings and discs in terahertz range. *Phys. Scr.* **94** (3), 035703 (2019).

[43] Liu, H., Wang, Z. H., Li, L., Fan, Y. X. & Tao, Z. Y. Vanadium dioxide-assisted broadband tunable Terahertz metamaterial absorber. *Sci. Rep.* **9** (2019). 10.1038/s41598-019-42293-9 PMC6453928 30962484

[44] Wen, Y., Ma, W., Bailey, J., Matmon, G. & Yu, X. Broadband terahertz metamaterial absorber based on asymmetric resonators with perfect absorption. *IEEE Trans. Terahertz Sci. Technol.* **5** (3), 406–411 (2015).

[45] Rahad, R., Mohsin, A. S., Bhuian, M. B. H. & Rahman, M. M. Graphene-metamaterial based tunable broadband polarization insensitive absorber for terahertz antenna design. *Ieee Access.* **12**, 48654–48667 (2024).

[46] Kong, X. et al. Transparent metamaterial absorber with broadband radar cross-section (RCS) reduction for solar arrays. *IET Microwaves Antennas Propag.* **14**(13), 1580–1586, (2020).

[47] Iwaszczuk, K. et al. Flexible metamaterial absorbers for stealth applications at terahertz frequencies. *Opt. Express*. **20** (1), 635–643 (2011). 10.1364/OE.20.000635 22274387

[48] Etman, A. S., Hameed, M. F. O., Obayya, S. S. A. & Hammad, A. E. Optimization of ultrathin polarization insensitive metamaterial absorbers using trust region algorithm based on Co-kriging model. *Opt. Mater.* **148**, 114823 (2024).

[49] Zhu, W. Electromagnetic metamaterial absorbers: from narrowband to broadband. *Metamaterials Metasurfaces*. (2018).

[50] Bagci, F. & Medina, F. Design of a wide-angle, polarization-insensitive, dual-band metamaterial-inspired absorber with the aid of equivalent circuit model. *J. Comput. Electron.* **16**, 913–921 (2017).

[51] Cai, H. et al. Multifunctional hybrid metasurfaces for dynamic tuning of terahertz waves. *Adv. Opt. Mater.* **6** (14), 1800257 (2018).

[52] Seo, M. et al. Active terahertz nanoantennas based on VO2 phase transition. *Nano Lett.* **10** (6), 2064–2068 (2010). 20469898 10.1021/nl1002153

[53] Zeybek, S. et al. Investigation of resonant properties of metamaterial THz filters fabricated from vanadium dioxide thin films. *Mod. Phys. Lett. B*. **38** (11), 2450056 (2024).

[54] Li, J. D. et al. Switchable VO2-metasurface for terahertz polarization converter and absorber. *Opt. Mater. Express*. **15** (9), 2079–2092 (2025).

[55] Wang, H. et al. Active dual-control Terahertz electromagnetically induced transparency analog in VO2 metasurface. *Appl. Phys. Lett.*, **123**(6). (2023).

[56] Chen, S., Lust, M., Roo, A. & Ghalichechian, N. Reliability of VO textsubscript 2-based MmWave switches under 100 million thermal cycles. *IEEE Trans. Device Mater. Reliab.* **23** (2), 241–248 (2023).

[57] Leroy, J. et al. High-speed metal-insulator transition in vanadium dioxide films induced by an electrical pulsed voltage over nano-gap electrodes. *Appl. Phys. Lett.*, **100**(21). (2012).

[58] Jiang, Z. et al. May. Flexible Terahertz metamaterials absorber based on VO2. In *Photonics*, Vol. 10, No. 6, 621 (MDPI, 2023).

[59] Althuwayb, A. A. et al. Design and performance evaluation of a novel metamaterial broadband THz filter for 6G applications. *Front. Mater.* **10**, 1245685 (2023).

[60] Papari, G. P., Pellegrino, A. L., Malandrino, G. & Andreone, A. Sensing enhancement of a Fabry-Perot THz cavity using switchable VO2 mirrors. *Opt. Express*. **30** (11), 19402–19415 (2022). 36221718 10.1364/OE.455941

[61] Elakkiya, A., Radha, S., Sreeja, B. S. & Manikandan, E. Terahertz broadband metamaterial absorber enabled by SiO 2, polyimide and PET dielectric substrates. *Pramana* **94** (1), 130 (2020).

[62] Jiao, X. F., Zhang, Z. H., Li, T., Xu, Y. & Song, G. F. Tunable dual broadband terahertz metamaterial absorber based on vanadium dioxide. *Appl. Sci.* **10** (20), 7259 (2020).

[63] Zhou, X. et al. Thermally tunable ultra-broadband terahertz metamaterial absorber with wide-angle impedance matching. *Int. J. Therm. Sci.* **220**, 110402 (2026).

[64] Hasan, R. & Anjum, N. *Design and analysis of a vanadium Dioxide-Based Ultra-Broadband Terahertz Metamaterial Absorber*. arXiv preprint arXiv:2508.05590 (2025).

